# Supplementary material for: Predictive Value of Measures of Vascular Calcification Burden and Progression for Risk of Death in Incident to Dialysis Patients
Source: J Clin Med. 2021 Jan 20;10(3):376. doi: 10.3390/jcm10030376 (PMC7863918; doi:10.3390/jcm10030376)
Supplement: Supplementary file 1 [file jcm-10-00376-s001.pdf]

Supplemental material

Table S1: comparisons between subjects enrolled in the Independent study with and without evaluation of abdominal aorta calcification (AAC) via the method described by Kauppila et al[10]

	Total (n=466)	Without Kauppila (n=282)	With Kauppila (n=184)	
**Variable**	**Mean (SD) or n (%)**	**Mean (SD) or n (%)**	**Mean (SD) or n (%)**	**P-Value**
**Age (years)**	**65.26 (14.78)**	**66.98 (13.83)**	**62.63 (15.8)**	**0.002**
Male sex (%)	49.57% (231)	48.58% (137)	51.09% (94)	0.664
Body weight (Kg)	69.63 (14.1)	70.47 (14.63)	68.34 (13.19)	0.105
**Diabetes (yes vs no)**	**29.61% (138)**	**34.75%(98)**	**21.74% (40)**	**0.004**
Systolic Blood pressure (mmHg)	136.01 (18.29)	136.39 (18.03)	135.42 (18.7)	0.579
Diastolic Blood pressure (mmHg)	75.84 (9.44)	75.81 (9.22)	75.9 (9.78)	0.923
**CAC (Agatston units)**	**283.41 (790.33)**	**92.51 (343.75)**	**575.98 (1124.04)**	**< 0.0001**
Pulse Wave velocity (m/sec)	9.18 (3)	8.96 (2.35)	9.52 (3.77)	0.07
QTc (msec)	408.96 (35.2)	406.99 (37.3)	411.98 (31.59)	0.121
QTd (msec)	28.31 (12.91)	27.15 (11.87)	30.09 (14.21)	0.021

Legend: CAC: Coronary Artery Calcification; QTc and QTd QT tract at electrocardiogram (EKG)

Table S2A: independent predictors of all-cause mortality in the study cohort 1. Variables forced into the model were selected based on available evidence and univariate analyses (table 1, variable with p<.15). Fully adjusted cox model (to predict all-cause of death): adjusted for Kauppila score, log(volume score) + PWV+ age + Framingham score + diabetes + ASCVD + Systolic Blood pressure + serum phosphate + serum calcium + serum sodium + serum PTH + use of ARBs + Use of betablockers +use of vitamin D + use of calcium based phosphate binder+ use of calcium channel blockers + use of cinacalcet.

Variable	HR	lower .95	upper .95	Pr(>|z|)
Kauppila score per 1 unit increase	0.9781	0.9192	1.041	0.4855
**log(volume score) per 1 log increase**	**1.8082**	**1.3486**	**2.424**	**<0.001**
**Pulse wave velocity (PWV) (m/sec)**	**1.0898**	**0.9979**	**1.19**	**<0.001**
Age (years)	1.0181	0.9841	1.053	0.2996
Framingham score (per 1 unit increase)	0.9825	0.8297	1.164	0.838
**Diabetes (yes vs no)**	**2.8998**	**1.2826**	**6.556**	**0.011**
ASCVD (yes vs no)	0.5691	0.2558	1.266	0.1671
Systolic blood pressure (mmHg)	1.0098	0.995	1.025	0.1953
Serum phosphate (mg/dl)	1.052	0.8625	1.283	0.617
Serum Calcium (mg/dl)	0.8909	0.6347	1.251	0.5044
Serum sodium (mEq/l)	1.0363	0.9486	1.132	0.4295

Parathyroid Hormone (iPTH) (pg/ml)	1.0004	0.9994	1.001	0.4272
Use of Angiotensin Receptor Blockers (ARBs) (yes vs no)	1.1667	0.4845	2.809	0.731
Use of beta-blockers (yes vs no)	0.8832	0.4863	1.604	0.6835
Use of vitamin D (yes vs no)	0.7688	0.4323	1.367	0.3708
Use of calcium based phosphate binder (yes vs no)	1.9395	0.5371	7.004	0.3119
**Use of calcium channel blockers (yes vs no)**	**1.7561**	**0.9514**	**3.241**	**0.072**
Use of cinacalcet (yes vs no)	1.0687	0.6232	1.832	0.8092

Table legend: ASCVD atherosclerotic cardiovascular disease defined if any of the following clinical data was reported: history of cerebrovascular disease; peripheral vascular disease; angina pectoris; history of myocardial infarction; aortic aneurysm; history of percutaneous coronary angioplasty with or without stenting; PWV pulse wave velocity; ARB: angiotensin receptor blockers; PTH: parathyroid hormone.

Table S2B: The most parsimonious model was then selected via a stepwise methods

Variable	HR	lower .95	upper .95	Pr(>|z|)
**log(CAC score) per 1 log increase**	**1.730**	**1.447**	**2.069**	**0.000**
**Pulse Wave velocity (m/sec)**	**1.0968**	**1.0082**	**1.193**	**0.03158**
Age (years)	1.0167	0.9967	1.037	0.10152
**Diabetes (yes vs no)**	**3.1042**	**1.4553**	**6.622**	**0.00338**
ASCVD (yes vs no)	0.5692	0.282	1.149	0.11584
Systolic blood pressure (mmHg)	1.0103	0.9966	1.024	0.13968
Use of calcium based phosphate binder (yes vs no)	2.6029	0.8045	8.421	0.11029
Use of calcium channel blockers (yes vs no)	1.6822	0.9516	2.974	0.07356

Table legend: CAC: coronary artery calcification; ASCVD atherosclerotic cardiovascular disease defined if any of the following clinical data was reported: history of cerebrovascular disease; peripheral vascular disease; angina pectoris; history of myocardial infarction; aortic aneurysm; history of percutaneous coronary angioplasty with or without stenting.

Reference

10 Kauppila, L.I.; Polak, J.F.; Cupples, L.A.; Hannan, M.T.; Kiel, D.P.; Wilson PW. New indices to classify location, severity and progression of calcific lesions in the abdominal aorta: A 25-year follow-up study. *Atherosclerosis* **1997**, *132*, 245–250.
